# Metabolic Complications Associated with Use of Integrase Strand Transfer Inhibitors (InSTI) for the Treatment of HIV-1 Infection: Focus on Weight Changes, Lipids, Glucose and Bone Metabolism

**DOI:** 10.1007/s11904-024-00708-x

**Published:** 2024-08-29

**Authors:** Stefano Savinelli, Ellen Newman, Patrick W. G. Mallon

**Affiliations:** 1 Department of Infectious Diseases, St Vincent’s University Hospital, Dublin, Ireland; 2 Centre for Experimental Pathogen Host Research (CEPHR), University College Dublin (UCD) School of Medicine, Dublin, Ireland

**Keywords:** HIV, InSTI, Weight gain, Lipids, Glucose, Bone health

## Abstract

**Purpose of Review:**

This review aims to summarize recently published peer reviewed papers on the influence of treatment with Integrase Strand Transfer Inhibitors (InSTI) in people with HIV (HIV) on metabolic health, including weight gain, lipid parameters, glucose homeostasis, and bone health.

**Recent Findings:**

InSTI have a mild/moderate effect on weight gain in both antiretroviral (ART) naïve and ART experienced PWH, which is more pronounced in certain groups (i.e. women, people of Black African ethnicity, those with lower socioeconomic status, and older people). The effect on weight is also driven by other components of the ART regimen as well as previous exposure to certain ART. InSTI have a relatively safe profile in terms of lipid parameters and bone health, compared to other ART classes, although some studies suggest a greater risk of insulin resistance and diabetes in PWH using InSTI, especially 2nd generation InSTI.

**Summary:**

While there is some evidence suggesting a negative impact of InSTI on some aspects of metabolic health (weight gain and glucose homeostasis), they remain the preferred treatment option for most PWH, due to their high efficacy and tolerability. However, an individualised approach to ART choice in PWH should be used in order to avoid negative outcomes in populations at higher risks of metabolic complications.

## Introduction

The natural course of HIV infection in the early years of the epidemic was characterized by unintentional weight loss and progressive decline in both lean and fat mass [1]. Initiation of antiretroviral therapy (ART) reverts the catabolic state associated with opportunistic infections and illnesses, resulting in restoration of body fat and protein stores. The subsequent weight gain is often referred to as return to health phenomenon, and has been associated with reduced mortality in people with HIV (PWH) after ART initiation [2]. Despite the health benefits associated with weight gain following ART initiation, concerns have been raised regarding excessive weight increases in PWH starting ART. In a large multicentre observational study from the USA and Canada, 22% of participants progressed from a normal body mass index (BMI) to the overweight category (BMI ≥ 25 kg/m^2^), and 18% from the overweight to the obesity category (BMI ≥ 30 kg/m^2^) over a follow-up period of 3 years after ART initiation, with the greatest weight gain observed the first year after starting ART [3]. More recently, an increasing body of evidence has linked the use of newer antiretrovirals like tenofovir alafenamide (TAF) and 2nd generation integrase strand transfer inhibitors (InSTI) to greater weight gain in PWH either starting ART or switching between ART regimens [4–6]. InSTI are currently recommended as first-line treatment for HIV-1 infection due to their high genetic barrier to resistance, rapid and sustained virological suppression, limited drug-drug interactions, and favourable safety profile [7]. In fact, evidence from randomised controlled studies shows a more favourable lipid profile in PWH treated with InSTI, as compared to protease inhibitors (PI) and non-nucleoside reverse-transcriptase inhibitors (NNRTI), both in those who are naïve to ART and in those switching to a different ART regimen [8]. Data from clinical trials also shows limited impact in terms of decline in bone mineral density (BMD) with the newer InSTI dolutegravir (DTG) and bictegravir (BIC) [9]. However, use of InSTI has also been associated with incident insulin resistance and diabetes in PWH starting ART, although with discordant results in different patient populations, with greater risk in people of African origin [10, 11]. The aim of this review is to briefly summarize the recent findings on weight gain, and alterations in lipids, glucose metabolism and bone health associated with the use of InSTI in PWH.

## Weight Gain Associated with InSTI Use in ART-naïve PWH

Studies on ART-naïve PWH offer valuable insights into the impact of specific classes of antiretrovirals on weight gain, as they enrol participants who were not previously exposed to ART. Although weight gain in ART-naïve PWH is expected as part of the return to health phenomenon, [2] these studies permit comparisons of weight change between ART classes and specific ART drugs and reveal distinct differences in weight change post ART between drugs.

In a large observational study from the Vanderbilt cohort enrolling 1,152 PWH starting ART, a substantial weight gain following initiation of ART was observed in the whole study population, irrespective of the antiretroviral class used (average weight gain 3.9 kg over 18 months) [12]. However, those starting DTG-based regimens had the highest average weight gain over 18 months (6 kg), which was significantly higher than that observed in those starting non-nucleoside reverse-transcriptase inhibitors (NNRTI, 2.6 kg) and the 1st generation InSTI elvitegravir (EVG, 0.5 kg). Weight gain associated with the use of raltegravir (RAL), another 1st generation InSTI, was also lower than that observed with DTG (3.4 kg), although the difference was not statistically significant.

Excess weight gain following ART initiation was particularly high in two open-label, multicentre, phase III, randomised controlled trials comparing DTG-based regimens with EFV-based ART as standard of care [13, 14]. The NAMSAL study compared DTG vs low-dose (400 mg) EFV for the treatment of HIV-1, both in combination with tenofovir disoproxil fumarate (TDF) and lamivudine (3TC). Median weight gain at 48 and 96 weeks was 5 kg in the DTG arm compared to 3 kg in the EFV arm (p < 0.001) [13, 15]. The ADVANCE study compared DTG, either in combination with TAF/emtricitabine (FTC) or TDF/FTC, against EFV/TDF/FTC. Mean weight gain was significantly higher (p < 0.001) in both DTG arms compared to the EFV arm, with the highest weight gain observed with TAF/FTC plus DTG (6 kg and 7.1 kg at weeks 48 and 96, respectively) [4, 14]. Similar findings were observed in the largest observational study to date from the NA-ACCORD cohort, a multicentre study enrolling participants in North America and Canada, where starting treatment with InSTI was associated with a mean weight gain of 4.9 kg and 5.9 kg over two and five years, respectively [16]. This was higher than the weight gain observed after initiation of NNRTI-based regimens (3.1 kg and 3.7 kg at two and five years, respectively), but it was not different from the weight change observed in participants starting PI-based regimens (4.9 kg and 5.5 kg at two and five years, respectively). Among the InSTI, the highest mean weight gain was observed with DTG (7.2 kg), while RAL and EVG were associated with smaller weight gains (5.8 kg and 4.1 kg, respectively). It is important to note that both RAL and EVG were mostly co-administered with TDF/FTC, while most of the participants on DTG were taking abacavir (ABC)/3TC as a backbone. This could have resulted in a smaller weight gain in people on RAL and EVG as a consequence of the known weight suppressive effect of TDF, which has also been confirmed in studies on HIV-negative participants [17]. Although there is more limited data available on BIC, the results of two randomised controlled trials have shown a substantial weight gain with BIC/FTC/TAF over 5 years (median weight change + 6.1 kg), with the greatest weight gains occurring in the first 3 years (+ 3 kg) [18].

An increasing number of observational studies in recent years have reported changes in weight and BMI associated with the use of InSTI in ART-naïve PWH, with weight gain ranging from + 1.93 kg to + 8.71 kg (Table 1) [19–25]. Multiple factors, not directly related to ART, could also explain some of the differences observed in published studies. Firstly, most of the studies report mean weight gain as opposed to median weight gain. Mean and median are two different measures of central tendency, and the presence of outliers could explain why mean weight change is usually greater than median weight change. Different sociodemographic factors could also contribute to the observed differences. Several studies have observed an association between weight gain following initiation of ART and factors like female sex, African ethnicity, older age, smoking and alcohol use [3, 26, 27]. Cultural factors, including dietary habits and perceptions around body image could also play a role in determining differences in weight change in PWH. For example, women of African origin from lower socioeconomic background or rural areas tend to prefer a BMI in the overweight range, rather than normal or underweight [28]. As per HIV specific factors, lower CD4+ T-cell count at baseline and more advanced HIV disease have been associated with greater weight gain following ART initiation [5, 25, 27]. While weight gain might be beneficial in PWH with advanced disease who are underweight, as part of a return to health phenomenon, PWH who have overweight or obesity prior to initiation of ART also tend to have a greater risk of > 10% weight gain and > 5 kg/m^2^ increase in BMI from baseline, which could lead increase the risk of development of cardiometabolic complications of obesity. In some studies, more than one third of PWH had either weight gain > 10% from baseline or treatment emergent obesity following initiation of InSTI based ART, even in those with a baseline BMI < 25 kg/m^2^ [20, 23, 25, 29]. On the other hand, other studies have shown either a modest weight gain following InSTI-based ART initiation [19, 24], or the absence of an increase in the rate of weight change following ART initiation, as compared to the pre-ART trend [30], although notably participants from these studies were predominantly male (65%-89%) and of white ethnicity (66%-84%), which may have influenced the study findings.

**Table 1:** Weight change in observational studies enrolling ART-naïve PLWH starting InSTI-based regimens

Study	Study design and details	Patient population	ART regimen	Weight change
Calza et al. 2019 [19]	Single centre observational study Country: Italy Years: 2013–2017	680 participants 84.3% Caucasian 69.1% male Mean age 42.1 years	RAL DTG EVG/c DRV/r	Mean weight change 12 months: RAL + 1.93 kg; + 0.71 kg/m^2^ BMI DTG + 2.38 kg; + 0.84 kg/m^2^ BMI EVG/c + 2.14 kg; + 0.77 kg/m^2^ BMI DRV/r + 1.85 kg; + 0.63 kg/m^2^ BMI
Galdamez et al. [20]	Single centre observational study Country: Spain Years: 2007–2019	219 participants Ethnicity not specified 77.8%-79.4% male Mean age 42.7–46.8 years	InSTI 24.7% Non-InSTI 75.3% (PI 52.1%, NNRTI 47.9%)	Mean weight change 96 weeks: InSTI + 4.1 kg NNRTI + 0.9 kg PI + 4.7 kg
Ando et al. [21]	Single centre retrospective study Country: Japan Years: 2005–2019	1579 participants 100% Asian 95.1% male Median age 37 years	InSTI 38.6% PI 58.8% NNRTI 2.5%	Mean weight change 5 years: InSTI + 3.8 kg PI + 3.5 kg NNRTI + 3.7 kg DTG highest weight change + 5.3 kg
Ruderman et al. [22]	Multicentre observational study Country: USA Years: 2012–2019	3232 participants 45% African American 16% female Mean age 37 years	InSTI 64% PI 12% NNRTI 24%	Weight change (linear mixed models) compared to EFV/FTC/TDF over 6 months: DRV/FTC/TDF + 3.7 kg DTG/FTC/TAF + 4.4 kg BIC/FTC/TAF + 3.9 kg
Bourgi et al. 2022 [23]	Multicentre observational study Country: Kenya Years: 2015–2018	17.044 participants 100% African 62% female Median age 36.8 years	NNRTI 97% DTG 3%	Mean weight change 18 months: DTG female + 6.1 kg DTG male + 4.1 kg NNRTI female + 2.6 kg NNRTI male + 2.8 kg
Nasreddine et al. [24]	Multicentre observational study Country: Belgium Years: 2019–2020	2001 participants 32.3% African 35.1% female 59.2% age < 50 years	BIC/FTC/TAF	Median weight change 48 weeks: + 3 kg
Grabar et al. [25]	Multicentre observational study Country: France Years: 2012–2018	12.773 participants 25.7% African 75.6% male Median age 38 years	InSTI 33.3% PI 41.7% NNRTI 25%	Mean weight change 30 months: Highest DTG + 8.71 kg Lowest EFV + 6.51 kg

Reference numbers as per main text. Abbreviations: *ART* Antiretroviral Treatment, *BIC* Bictegravir, *BMI* Body Mass Index, *DRV/r* Darunavir/ritonavir, *DTG* Dolutegravir, *EFV* Efavirenz, *EVG/c* Elvitegravir/cobicistat, *FTC* Emtricitabine, *InSTI* Integrase Strand Transfer Inhibitor, *NNRTI* Non-Nucleoside Reverse-Transcriptase Inhibitor, *PI* Protease Inhibitor *RAL* Raltegravir, *TAF* Tenofovir Alafenamide, *TDF* Tenofovir Disoproxil Fumarate

In summary, initiation of ART with InSTI-based regimens in ART-naïve PWH has been associated with variable effects on weight gain, with a higher risk of weight gain in females, people of African ancestry, older age, those with more advanced disease and those with either underweight or overweight and obesity pre-ART. Outside of these “risk categories” weight gain is generally modest. Clinical implications of excess weight gain following ART initiation are still unclear.

## Weight Gain Associated with InSTI in PWH Switching from a Different ART Class

Studies of virologically suppressed PWH on stable ART switching to InSTI-based regimens provide the opportunity to assess the impact of InSTI use on weight gain in the absence of return to health phenomenon that accompanies ART initiation. Table 2 summarizes reported absolute weight changes, weight and BMI trajectories following switch to InSTI-based regimens from recent RCT and observational studies.

**Table 2:** Weight change following switch to InSTI-based regimens in ART-experienced PLWH

Study	Study design and details	Patient population	ART regimen	Weight change
Norwood et al. [31]	Single centre observational study Country: USA Years: 2005–2015	495 participants 38–46% Non-white 14–29% female Median age 38.5–39.7 years	Baseline ART: TDF/FTC/EFV Switch ART: 27.5% InSTI 6.9% PI 65.6% kept baseline ART	Mean weight change 18 months: TDF/FTC/EFV + 0.8 kg InSTI + 2.9 kg PI + 0.7 kg Greatest weight gain with ABC/3TC/DTG + 5.3 kg vs + 2.8 kg with RAL or EVG/c
Kerchberger et al. [32]	Multicentre observational study Country: USA Years: 2006–2015	1118 participants 61% African American All female Mean age 48.8 years	Baseline ART: NNRTI 49.6% PI 51.2% Switch ART: DTG 42% EVG/c 23% RAL 36%	Mean weight change 2 years: Switch to InsTI + 2.4 kg; + 0.9 kg/m^2^ BMI Remained on non-InSTI + 0.2 kg; + 0.1 kg/m^2^ BMI
Leonard et al. [33]	Two retrospective multicentre cohorts Country: USA Years: not specified	101 participants 26.7–34.2% Black 82.5–83.2% male Median age 38–53.6 years	Baseline ART: EFV-based ART Switch ART: DTG 57.4% EVG/c 33.7% RAL 8.9%	Median weight change 48 weeks: White + 1.8 kg Black + 2.8 kg Male + 2.2 kg Female + 0.7 kg
Llibre et al. [34]	Multicentre RCT Countries: North America, Europe, South America, Asia, South Africa Years: 2019–2021	493 participants 19% African heritage 39% female Median age 45 years	Baseline ART: 50% NNRTI 40% InSTI 10% PI Switch ART: 3TC/DTG	Mean weight change 48 weeks: 3TC/DTG + 2.1 kg; + 0.7 kg/m^2^ BMI Continuation of baseline ART + 0.6 kg; + 0.2 kg/m^2^ BMI
Osiyemi et al. [35]	Multicentre RCT Countries: North America, Europe, Asia, Oceania Years: 2018–2021	741 participants 14–16% Black/African American 7–9% female Median age 39–40 years	Baseline ART: 78–80% TAF + InSTI 13–14% TAF + NNRTI 8% TAF + PI Switch ART: 3TC/DTG	Mean weight change 144 weeks: 3TC/DTG + 2.2 kg Continuing TAF-based baseline ART + 1.7 kg
Esber et al. [36]	Multicentre observational study Country: Kenya, Tanzania, Nigeria, Uganda Years: 2013–2020	3524 participants All Black African 57.8% female Median age 38 years	Baseline ART: 73.9% TDF/3TC/EFV 23.1% AZT/3TC/EFV < 1% PI Switch ART: TDF/3TC/DTG	Median weight change 1st year: Switch to TDF/3TC/DTG + 1.36 kg; + 0.68 kg higher than those who remained on baseline ART Switch to TDF/3TC/DTG + 0.44 kg/m^2^ BMI
Waters et al. [37]	Multicentre RCT Countries: Belgium, France, Germany, Italy, Spain, UK Years: 2014–2017	412 participants 85% White 89.1% male Median age 54 years	Baseline ART: PI-based (51.8% DRV/r), 65.2% TDF/FTC Switch ART: DTG + same NRTI	Mean weight change 48 weeks: + 0.8 kg following switch to DTG % of participants experiencing > 5% weight gain + 9.9% at 48 weeks
Brennan et al. [38]	Single centre observational study Country: South Africa Years: 2010–2020	6.948 participants Mostly Black African 51.5% male Median age 38.8 years	Baseline ART: TDF/XTC/EFV Switch ART: 11.4% DTG (maintaining TDF/XTC) 88.6% kept baseline ART	Mean weight change 12 months: TDF/XTC/EFV + 1.5 kg (+ 2.2%) DTG + 2.8 kg (+ 4.5%)
Goldberg et al. [39]	Single centre observational study Country: USA Years: 2011–2018	103 participants Incarcerated males 86% African American Mean age 44 years	Baseline ART: TDF-based NNRTI or PI Switch ART: 40% EVG/c 29% DTG 18% RAL 13% BIC 1/3 on TDF/FTC, 1/3 on TAF/FTC, 1/3 on ABC/3TC	Mean weight change 12 months: Switch to InSTI + 4.9 kg; + 1.6 kg/m^2^ BMI Switch to DTG highest weight gain + 6.5 kg; + 2.8 kg/m^2^ BMI Switch to BIC + 4.9 kg Switch to RAL lowest weight gain + 3.6 kg Higher weight gain in those on TAF/FTC (+ 6.4 kg) vs TDF/FTC (+ 2.1 kg)
Verburgh et al. [40]	Multicentre observational cohort study Country: Netherlands Years: 1998–2020	5091 participants 71.8% Caucasian 84.3% male Median age 49.3 years	Baseline ART: TAF- and InSTI-naïve Switch ART: 30.4% TAF only 51.6% InSTI only 18% InSTI + TAF	% of participants with ≥ 10% weight gain at 24 months: 8.8% TAF; 10.6% InSTI; 14.4% InSTI + TAF Absolute mean weight change 24 months in those with ≥ 10% weight gain: + 9.38 kg TAF + 9.59 kg InSTI + 10.24 kg InSTI + TAF
Lake et al. [41]	Two multicentre observational cohorts Country: USA Years: 1997–2017	972 participants 50% non-White 81% male Median age 50 years	Baseline ART: NNRTI 31% PI 68% Other non-InSTI 5% Switch ART: RAL 55.4% EVG/c 22.8% DTG 21.8%	Annualized weight change: Pre-switch + 0.4 kg/year Post-switch + 0.6 kg/year White or Black race, age ≥ 60 years, BMI ≥ 30 kg/m^2^ greater annualized weight gain (+ 0.9 to + 2 kg/year)
Verboeket et al. [42]	Single centre observational cohort study Country: Netherlands Years: 2010–2018	605 participants 7–11% African ethnicity 83–91% male Median age 53–55 years All ≥ 45 years	Baseline ART: 45.4% NNRTI 49.6% PI 5% other non-InSTI Switch ART: 53% DTG 34% EVG/c 13% RAL	Weight trajectories pre- and post-switch over median 1.9 years: + 0.15 kg/year pre-switch; + 0.14 kg/year post-switch Probability of weight gain > 5% baseline higher in InSTI switch (23.1%) vs non-switch (13.2%) vs HIV-negative controls (15.7%)
Mallon et al. [6]	Multicentre observational cohort study Country: USA Years: 2010–2019	1429 participants (InSTI switch) 38% Black 18% female Median age 49 years	Baseline ART: 74.2% PI 25.8% NNRTI All on TDF Switch ART: TAF + InSTI: 78% EVG/c 12% DTG	Weight gain per year over 9 months: EVG/c + 2.55 kg/year DTG + 3.09 kg/year BIC + 4.47 kg/year

Reference numbers as per main text. Abbreviations: *3TC* lamivudine, *ABC* abacavir; *ART* Antiretroviral Treatment, *BIC* Bictegravir, *BMI* Body Mass Index, *DRV/r* Darunavir/ritonavir, *DTG* Dolutegravir, *EFV* Efavirenz, *EVG/c* Elvitegravir/cobicistat, *FTC* Emtricitabine, *InSTI* Integrase Strand Transfer Inhibitor *NNRTI* Non-Nucleoside Reverse-Transcriptase Inhibitor, *PI* Protease Inhibitor, *RAL* Raltegravir, *RCT* randomised controlled trial, *TAF* Tenofovir Alafenamide, *TDF* Tenofovir Disoproxil Fumarate, *XTC* lamivudine or emtricitabine

Absolute weight change following switch to InSTI-based ART was generally modest in most studies, ranging from + 0.8 kg to + 2.9 kg over an observation period of 48 weeks to 2 years following switch [31–38]. Two observational studies reported substantially greater weight gain following switch to InSTI, with up to + 6.5 kg weight gain in PWH from a correctional facility switching to DTG [39], and up to + 10.24 kg in a Dutch cohort of PWH switching to TAF + InSTI [40]. Interestingly, studies looking at weight trajectories prior to and after switch to InSTI showed either a small increase in annualized weight gain (+ 0.6 kg/year post-switch vs + 0.4 kg pre-switch) [41], or no difference in weight change per year before and after switch (+ 0.14 kg/year post-switch vs + 0.15 kg/year pre-switch) [42]. However, in stark contrast with the above mentioned studies, the results of the OPERA cohort showed a marked difference in annualized weight change in those switching from TDF-based regimens (+ 0.01 kg/year to + 0.24 kg/year pre-switch) to TAF + InSTI (+ 2.55 kg/year to + 4.47 kg/year in the first 9 months post-switch) [6].

Several factors could contribute to the observed differences between studies, including:

1. Sociodemographic factors
  Greater weight change following switch to InSTI was generally reported in studies with a higher representation of people of African ancestry [31–33, 39], as opposed to studies enrolling predominantly White men who have sex with men (MSM) [37]. In this regard, data from the ATHENA cohort from the Netherlands showed that females from western and sub-Saharan Africa were at increased risk of weight gain ≥10% of baseline weight following switch to InSTI compared to western males [40]. Interestingly, this was the study reporting the highest mean weight change following switch to InSTI (+9.59 kg in those switching to InSTI only, and +10.24 kg in those switching to InSTI + TAF). The possible influence of ethnicity on weight gain following switch to InSTI is also suggested by the results of a small, single centre observational study enrolling incarcerated men living with HIV of predominantly African American background (86%), where switch to InSTI was associated with a mean weight gain of +4.9 kg over 12 months, with the highest weight gain in those switching to DTG (+6.5 kg) [39]. While no formal analysis was conducted in this study on factors associated with weight gain, it is unlikely that lifestyle changes (i.e. diet and exercise) could have contributed to weight gain in a strictly controlled environment, and the high representation of African American men in this study might explain the observed greater weight gain. Finally, some studies have suggested that older age groups of PWH might not experience a significant weight gain following switch to InSTI, in particular DTG [42, 43]. However, data from a multicentre observational study from the US showed that older age was associated with greater rates of BMI increase following switch to InSTI [44]. Differences in study setting and population characteristics might explain the contrasting results on the role of age in contributing to weight gain in PWH switching to InSTI.
2. ART regimen prior to switch
  Differences in NRTI backbone and third agent prior to switch to InSTI-based regimens have been shown to result in a variable degree of weight change, both in RCT and observational studies. Greater weight gain was observed in PWH who switched from TDF-based regimens to different NRTI backbones. In the Vanderbilt cohort, the highest mean weight gain (+5.3 kg over 48 weeks) was observed in those who switched from TDF/FTC/EFV to ABC/3TC/DTG [31]. Similar effects of switching away from TDF were observed even with dual drug regimens, as shown by the analysis of adverse events from the SALSA trial, where ART-experienced PWH on TDF-based regimens had a greater weight gain than those who were not on TDF (+2.4 kg vs +1.8 kg, respectively) following switch to 3TC/DTG [34]. The importance of the NRTI backbone in driving weight gain has been emphasized by studies looking at the effect of switching to a combination of TAF + InSTI, where more extreme weight changes were observed compared to those who were using InSTI with a different NRTI backbone [40], especially when TDF was used prior to switch [6].
  Notwithstanding the important contribution of TDF and TAF to weight dynamics in PWH switching to InSTI-based regimens, there is also evidence supporting an independent effect of InSTI themselves in driving weight gain. In the AFRICOS cohort, ART-experienced PWH switching to TDF/3TC/DTG had a mean weight gain of +1.36 kg over the first year, compared to an annual increase of 0.35 kg/year prior to switch [36]. Most of the participants (73.9%) were on TDF/FTC/EFV prior to switch, suggesting that even in those who remain on TDF, switching to DTG could still contribute to substantial weight gain. Similar results were also observed in a recent observational study from South Africa, where switching to DTG while maintaining the same TDF-based backbone was associated with greater weight gain than maintaining baseline ART (+2.8 kg vs +1.5 kg ) [38]. In a large multicentre observational study from the US that analysed BMI trajectories, although PWH who switched to TAF + InSTI had a more rapid BMI change than other groups, switch to InSTI remained associated with an accelerated BMI change irrespective of the use of TAF when comparing BMI slopes prior to switch to those after switch [44]. Interestingly, switching from TAF-based regimens to 3TC/DTG in the TANGO trial resulted in similar weight changes in the two groups (+1.7 kg in those who remained on TAF versus +2.2 kg in the 3TC/DTG switch group) over 144 weeks [35].
  What role, if any, is played by the third agent (NNRTI or PI) prior to switch to InSTI in determining future weight gain is less well established. In two large multicentre observational studies from the USA, switching from NNRTI to DTG and EVG/c was associated with increased annualized weight gain, while switching from PI only resulted in greater weight gain only when switching to DTG [41]. This is in line with data from a pooled analysis of 12 RCT of PWH on suppressive ART switching to different regimens, where switching off EFV was an independent predictor of subsequent weight gain, especially when switching to InSTI-based regimens [45]. Greater weight gain following switch off EFV could be related either to a potential weight suppressive effect of EFV or to detrimental effects of other third agents (e.r. PI) on weight that mask any subsequent impact of InSTI. As EFV is metabolised through the cytochrome P450 2B6 pathway, CYP2B6 slow metabolisers who are exposed to higher circulating levels of EFV, may experience enhanced weight suppression while on EFV and then experience greater weight gain when switching off EFV [33]. On the other hand, in a post hoc analysis of the NEAT-022 trial, which examined weight changes following a PI to InSTI switch while maintaining the same NRTI backbone, weight gain over 48 weeks after switch was only marginal (+0.8 kg), again suggesting the possible influence of pre-switch PI-based ART on weight that impact further weight change following switch to InSTI [37]. This is also supported by data from the ATHENA cohort, where discontinuation of EFV when compared to discontinuing atazanavir/ritonavir (ATV/r), was independently associated with increased risk of weight gain >10% above baseline weight [40]. In conclusion, while these data point towards a role of the third agent prior to switch to InSTI in determining greater weight gain, InSTI themselves and DTG in particular are associated with mild/moderate weight gain in virologically suppressed PWH switching from different ART classes.
3. Type of InSTI
  While all InSTI have been associated with some degree of weight gain in PWH switching from other ART regimens, the 2^nd^ generation InSTI, DTG and BIC, have been associated with greater weight gain than 1^st^ generation InSTI, RAL and EVG/c in most published studies [6, 31, 39]. Within the InSTI class, DTG has been associated with the highest rates of weight gain, while more limited data is available on BIC, given the smaller number of participants enrolled in studies published so far. The proposed mechanisms behind greater weight gain associated with 2^nd^ generation InSTI use will be discussed later in this review.
4. Baseline Weight and BMI
  Lower BMI prior to switch has been associated with greater subsequent weight gain post switch in an observational of PWH from South Africa switching from EFV- to DTG-based regimens [38]. The opposite was observed in a multicentre observational study from the US, where PWH with a higher BMI prior to switch had greater BMI changes following switch to InSTI [44]. This discrepancy might have been influenced by differences between study populations, but it could also be related to the weight suppressive effect of EFV discussed above [33], given that all PWH from the first study were on EFV-based regimens thus explaining greater weight gain following switch from EFV to DTG.

Finally, it is important to note that weight gain following switch to InSTI seems to be mainly limited to the first 8–9 months following switch [6, 44], while long-term use of InSTI has not been associated with greater change in BMI compared to PWH on non-InSTI regimens [46]. However, it is still unclear whether the short-term weight gain experienced in the first few months after switch to InSTI could be reversible and whether it is associated with health consequences, as discussed later in this review.

In summary weight gain following switch to InSTI-based ART is a complex phenomenon that is influenced by multiple factors, including age, sex, ethnicity, pre-switch third agent, and possibly starting BMI. However, weight gain following switch to InSTI has also been observed in cohorts with a low prevalence of potential “risk factors” for weight gain [42], suggesting that some PWH might experience excessive weight gain following switch to InSTI in the absence of known contributory factors, highlighting the need for an individualised approach to metabolic health in PWH.

## Weight Gain with InSTI Use for Pre-exposure Prophylaxis (PrEP)

Studies on PrEP offer an interesting perspective to explore the impact of ART on weight gain in the absence of HIV infection. Cabotegravir (CAB), a 2nd generation long-acting InSTI, is the first InSTI to be studied in PrEP trials [47]. Although it is still not widely available, and real-world data on its use are lacking, in the HPTN 083 trial comparing CAB with TDF/FTC for PrEP in HIV-negative participants, a greater mean annualized increase in weight was observed in the CAB group compared to the TDF/FTC group (+ 1.23 kg/year vs + 0.37 kg/year, respectively) [47]. Consistent with weight changes observed with InSTI in PWH, excess weight gain with CAB as PrEP was limited to the first 40 weeks of the study, and rates of weight change rates did not differ between the two groups later in the trial. In contrast, use of CAB in the HPTN 077 trial, which compared CAB versus placebo for PrEP in HIV-negative participants, was not associated with greater weight gain over 41 weeks (+ 1.1 kg median weight gain with CAB vs + 1.0 kg with placebo) [48]. While the small sample size (146 participants) should prompt caution in interpreting the results of this trial, the differences between the two studies might be explained by the proposed weight suppressive effect of TDF rather than by a specific effect of CAB on increasing weight. However, as noted above, TDF use was not associated with reduction in weight in HPTN 083. It is important to point out that there are currently no studies published in peer-reviewed journals on metabolic effects of CAB for the treatment of HIV-1 infection in PWH.

## Mechanisms of Weight Gain Associated with Use of InSTI

An increasing number of studies in recent years have explored potential pathophysiological mechanisms underlying weight gain with ART, with a focus on InSTI. The most widely studied mechanisms in recent years will be briefly summarised below:

1. Inhibition of the melanocortin 4 receptor (MC4R). InSTI have been shown to antagonize the activity of the MC4R receptor, which is involved in the regulation of body weight [49]. Inhibition of binding of endogenous ligands to MC4R results in increased food intake and weight gain. While all InSTI were able to interfere with the MC4R function in experimental models, it should be noted that this occurred at drug concentrations which were substantially greater than those achieved in clinical practice [49]. The relative role of MC4R in driving weight gain with InSTI in vivo remains unclear.
2. Alterations in thermogenesis and mitochondrial function. In mice, DTG can reduce energy expenditure and thermogenesis in brown and beige adipose tissue by downregulating uncoupling protein 1 (UCP1), and can also decrease mitochondrial respiratory capacity, possibly contributing to excess weight gain [50]. In addition, some individuals might have a greater predisposition towards weight gain with the use of InSTI and/or TAF as a consequence of polymorphisms in mitochondrial DNA (mitochondrial haplogroups) associated with alterations in energy expenditure and metabolism [51].
3. Alterations in adipose tissue function. In vitro studies on human, primate and mice have shown that InSTI, especially DTG, exert pro-fibrotic effects and induce adipocyte hypertrophy in both subcutaneous and visceral adipose tissue, with alterations in adipose tissue metabolism and function possibly contributing to weight gain [52]. Studies on human adipocytes have also shown a direct influence of DTG and BIC on production of adipokines like adiponectin and leptin, as well as inflammatory cytokines within the adipose tissue, with subsequent alterations in adipose tissue homeostasis promoting insulin resistance [53].

However, the exact mechanisms underlying weight gain associated with InSTI use in PWH and the possible explanations for excess weight gain in people with specific risk factors remains unclear.

## Clinical Implications of Weight Gain in PWH Treated with InSTI

As discussed in the previous paragraphs, use of InSTI is associated with a variable degree of weight gain, from mild/moderate in most cases to substantial increase in weight in a smaller proportion of PWH. However, an important question which still remains unanswered is how much weight gain is clinically significant?

A weight increase of > 5% of baseline weight is generally considered to be clinically relevant. This is derived from the Federal Drug Administration (FDA) guidelines for the evaluation of clinical trials for weight loss, where a weight loss of 5% of starting weight is considered clinically meaningful [54], while weight gain of > 10% has been associated with increased risk of cardiovascular events in epidemiological studies in the general population [55].

Only a few studies have explored the clinical consequences of weight gain in PWH. In a single centre observational study from South Africa enrolling predominantly PWH of black African ancestry, switching from EFV to DTG was associated with an increased risk of hypertension (risk difference 14.2% for DTG vs EFV) [38]. In the same cohort, switching to DTG was also associated with greater weight gain compared to staying on EFV, although the mean weight change was less than 5% (+ 4.5% with DTG vs + 2.2% with EFV). In a retrospective analysis of a large healthcare database from the USA, ART-naïve PWH starting InSTI were at significantly higher risk of developing congestive heart failure, myocardial infarction, and lipid disorders, compared to PWH starting PI and NNRTI based regimens [56]. However, data on weight was not reported in the study, therefore it is not possible to speculate on the association between weight gain and cardiometabolic outcomes observed in this population. In an analysis of the NA-ACCORD cohort of 22,884 PWH starting ART, initiation of InSTI was associated with a 17% higher risk of incident diabetes over 12 months, compared to NNRTI, with the highest risk observed with RAL [57]. In this study, the effect of InSTI on incident diabetes was attenuated when adjusting for weight gain over 12 months, suggesting that most of the excess risk of diabetes was attributable to weight gain. However, this was not observed when considering DTG and EVG separately, suggesting that these antiretrovirals could have a direct influence on insulin resistance and diabetes independent of weight gain.

Another interesting aspect to consider when assessing the consequences of weight gain with the use of InSTI is whether weight gain is also accompanied by concomitant alterations in body fat mass and distribution. In a single centre, retrospective, observational study from Italy, PWH switching to InSTI experienced a threefold increase in subcutaneous adipose tissue area and a sevenfold increase in the visceral adipose tissue area as assessed by CT scan over a median of 36 months, compared to those who did not switch to InSTI [58]. However, rates of BMI increase over time were similar between the two groups, suggesting that while InSTI could have an impact on body fat distribution, with increase in both subcutaneous and visceral adipose tissue, this might not be directly associated with weigh gain.

In summary, negative clinical consequences of weight gain associated with the use of InSTI have been reported, although further prospective studies are needed to understand the clinical significance of weight gain versus alterations in body fat composition in PWH treated with InsTI.

## Impact of InSTI on Lipid Parameters

Given the increasing number of studies showing an association between InSTI use and weight gain, there is growing interest in the potential adverse effects of InSTI on metabolic health in PWH. Despite the concerns around weight gain, most of the published studies show a favourable lipid profile in PWH treated with InSTI. In a multicentre, observational study from Spain enrolling 348 ART experienced PWH, switch to DTG/rilpivirine (RPV) was associated with significant reductions in levels of total cholesterol, LDL and triglycerides over 48 weeks [59]. Most of the study participants were on PI and NNRTI based regimens prior to switch, and the main reasons for switch were either convenience or toxicity.

In another multicentre trial evaluating the efficacy and safety of a InSTI-based dual drug regimen including RAL in combination with etravirine (ETR), virologically suppressed PWH switching from PI based regimens also experienced significant reductions in levels of proatherogenic lipids over 48 weeks, with a decrease in total cholesterol of 0.5%, non-HDL cholesterol of 4.6%, LDL cholesterol of 4.3%, triglycerides of 18.8%, while HDL cholesterol increased by 5.4% at week 48 [60]. All changes were sustained up to week 96, and the favourable effect on lipid profile was observed not only in PWH with a diagnosis of dyslipidaemia (53% of study participants), but also in those without dyslipidaemia.

The effect of initiation of InSTI based regimens on lipid parameters in ART naïve PWH was summarized in a recent systematic review and meta-analysis of 6 RCTs including a total of 3,521 PWH [61]. Five of the included studies showed a significant reduction in total cholesterol and triglycerides, while three studies showed a reduction in LDL over observation periods between 48 and 96 weeks, again suggesting a favourable impact of InSTI on lipid parameters.

These “lipid-friendly” properties of InSTI in PWH were also observed in studies comparing InSTI based regimens with other ART classes. In the RESPOND cohort, a multicentre, prospective, collaborative study across Europe and Australia, the incidence of dyslipidaemia was compared between PWH on INSTI vs boosted PI vs NNRTI [62]. Of 4,577 PWH included in the analysis (42.3% naïve to ART), 1,460 (32%) participants developed dyslipidaemia during 7,621.3 person-years of follow-up. Use of InSTI was associated with a 29% lower chance of developing hyperlipidaemia compared to boosted PI.

Similar findings were described in a single-centre, retrospective study comparing weight gain in ART naïve PWH started on DRV/r vs InSTI, where there were no significant changes over 12 months in total cholesterol, LDL and HDL in those starting InSTI, while PWH starting DRV/r had a significant increase in total cholesterol, LDL and triglycerides [19].

On the other hand, use of InSTI in the RESPOND study was associated with a 35% greater chance of developing dyslipidaemia than NNRTI based regimens [62], suggesting that NNRTI might be associated with a more favourable lipid profile than InSTI.

Studies showing a worsening lipid profile associated with the use of InSTI are mostly limited to those where InSTI were used in combination with TAF. In the ATHENA cohort, significant increases in non-fasting total cholesterol, LDL, and triglycerides were only seen in ART-experienced PWH who switched to TAF based regimens, including those who switched to TAF plus InSTI, while those who switched only to InSTI did not experience worsening dyslipidaemia [40]. Switching from a TAF based regimen to DTG/3TC was associated with a more favourable lipid profile at 96 and 144 weeks in the TANGO study, suggesting a predominant influence of TAF on dyslipidaemia in PWH treated with modern ART regimens [35]. Interestingly, incident use of lipid-lowering agents from baseline was not different in the DTG/3TC arm, compared to those remaining on TAF based regimens, suggesting possible lingering effects of TAF on lipid abnormalities, which are not resolved with the use of InSTI. In contrast to the above mentioned data, switch to TAF/FTC/BIC in virologically suppressed PWH over the age of 50 years was associated with a significant decrease in total cholesterol, LDL and triglycerides over 48 weeks, despite the fact that 20% of participants were on TDF based regimens prior to switch [63]. However, most of the participants (55%) were on InSTI based regimens prior to switch and only 13% were on PI based regimens, which have been associated with worse lipid profiles in other studies.

Finally, data on the effect of newer generation InSTI like cabotegravir (CAB) are mostly limited to its use in HIV negative people in the context of PrEP, were use of CAB has been associated with a neutral effect on lipid parameters [48].

## Impact of InSTI on Glucose Metabolism

Another important aspect of metabolic health which is increasingly being studied in PWH on ART is glucose metabolism. Use of older ART like thymidine analogues and didanosine has been associated with insulin resistance in PWH [64]. In more recent years, an increasing number of studies have focused on alterations in glucose metabolism in PWH treated with InSTI, with a special focus on its association with weight gain.

In an analysis of the Women Interagency HIV Study, women living with HIV who switched to an InSTI based regimen were compared to those who remained on a non-InSTI based regimen and followed up for a median of two years [65]. Women who switched to InSTI experienced a significantly higher increase in HbA1c over 6–18 months compared to those who remained on non-InSTI based regimens (+ 0.05% vs -0.06%). When data were stratified by weight gain, the greatest increase in HbA1c was observed in those switched to InSTI who had a clinically meaningful increase in weight of > 5% above baseline weight (+ 0.3%), even compared to those who did not switch to InSTI but also experienced a similar weight gain over the same observation period (-0.04%). In contrast, change in HbA1c following switch to InSTI did not differ from those who remained on non-InSTI based regimens in the group who gained < 5% of baseline weight, suggesting the presence of an association between weight gain and worsening glycemic control associated with the use of InSTI.

When looking at the incidence of new onset diabetes following initiation of ART, use of InSTI was not associated with a greater risk of diabetes mellitus in a large multicentre study from France enrolling PWH starting ART between 2009 and 2017 [66]. Of the 19,462 participants, new onset diabetes was diagnosed in 265 PWH (1.36%) over a median follow-up time of almost 2 years. Interestingly, the incidence of diabetes did not differ between those started on InSTI (0.91%), compared to those starting NNRTI (1.37%) or PI (1.5%). Only BMI > 30 kg/m2, age older than 37 years, black race or Hispanic ethnicity, hypertension and AIDS were independently associated with incident diabetes in adjusted analyses. However, it is important to note that only 35% of PWH on InSTI were using DTG and < 1% were on BIC, limiting the generalizability of the results to 2nd generation InSTI.

Other studies focusing on 2nd generation InSTI and DTG in particular, have shown a greater risk of hyperglycaemia, insulin resistance and diabetes mellitus associated with their use in PWH. In a multicentre study from Zambia, use of DTG was among the factors associated with a higher risk of metabolic syndrome in PWH, which was defined based on the levels of fasting glucose, HDL, triglycerides, systolic and diastolic blood pressure measurements, and waist circumference [67]. In particular, PWH on InSTI had a twofold higher risk of metabolic syndrome than those on NNRTI based regimens. Similarly, in a case–control study from Uganda specifically looking at risk of hyperglycaemia in PWH on DTG containing regimens, prior use of DTG was associated with seven times higher odds of developing hyperglycaemia compared to those whose previous ART did not include DTG [68]. The hyperglycaemia observed in this study was of clinically significant magnitude, given that more than 70% of the study participants were diagnosed with diabetes mellitus over the study period.

However, in other studies the risk of diabetes associated with use of InSTI was not only limited to DTG, but was also observed with 1st generation InSTI. In an analysis of two large databases from the US looking at ART naïve PWH starting ART between 2007 and 2019, use of DTG, EVG/c and RAL was associated with a greater risk of hyperglycaemia and type 2 diabetes mellitus, with the highest risk associated with the use of EVG/c (HR 1.54), followed by DTG (HR 1.26), and RAL (HR 1.19) [10]. Interestingly, BIC was not associated with a significantly higher risk of hyperglycaemia/diabetes, although only a small number of PWH (5.7% of those on InSTI) were on BIC based regimens. Although data on the impact of BIC on glucose metabolism are limited, cases of hyperglycaemia and ketoacidosis following initiation of BIC based regimens in PLWH have been reported in the literature [69].

While the mechanisms behind the increased risk of hyperglycaemia and diabetes in PWH using InSTI are not well known, it is hypothesized that InSTI could interfere with insulin release and signalling by chelation of circulating magnesium [70].

## Impact of InSTI on Bone Health

Among the age-related complications, low bone mineral density (BMD) leading to osteopenia and osteoporosis is highly prevalent in PWH, who also have a higher risk of fractures than the general population [71]. However, most of the studies showing an increased risk of fractures in PWH were conducted prior to the wider availability of InSTI in clinical practice [72]. Several studies have indicated that InSTI based regimens have a safer bone profile compared to older ART. For example, in a multicentre, observational study of ART experienced PWH over the age of 45 years switching from a PI based regimen to a dual drug regimen of RAL plus ETR, there was a significant increase in BMD at week 48 (+ 0.7% in lumbar spine BMD) following switch to InSTI based regimen [60].

The mechanisms behind the safer bone profile of InSTI compared to PI are not fully understood. In a small observational study enrolling 30 PWH treated with PI or RAL, markers of osteoblast function were measured at baseline and after 1 year of ART [73]. Serum levels of undercarboxylated osteocalcin and pentosidine, both markers of poor bone health associated with higher risk of fractures, were significantly higher in PWH after 1 year of treatment with PI compared to those treated with RAL. Ten participants were switched from PI to RAL based regimens, and the authors observed a significant reduction in both undercarboxylated osteocalcin and pentosidine 6 months after switching, suggesting an improvement in osteoblast function associated with use of RAL compared to PI.

Osteopenia and osteoporosis are particularly relevant to women living with HIV who, like the general population, experience issues with bone health at a younger age than men. In an analysis of the Modena HIV cohort, women living with HIV had a faster decline in BMD over time compared to men [74]. Reasurringly, a longer history of exposure to InSTI was independently associated with higher femoral and lumbar spine BMD, although it is important to note that only 199 of 2,596 participants were on InSTI based regimens, almost all of whom were on RAL.

Second generation InSTI have also been shown to have a less negative impact on bone health. In the SALSA trial, PWH switching to DTG/3TC had a significant reduction in markers of bone turnover (bone specific alkaline phosphatase, osteocalcin and pro-collagen 1 N-terminal propeptide) compared to those continuing 3 or 4 drug regimens [34]. However, 40% of participants were already on an InSTI based regimen prior to switch, and 44% were on TDF. Therefore, the improvement in markers of bone health could have been influenced by discontinuation of TDF, which is known to be accompanied by improvements in BMD and reductions in bone turnover markers.

BIC has also demonstrated good safety profile concerning bone health in women living with HIV. An integrated analysis encompassing five trials involving women of various ages, both ART-naïve and experienced, revealed relatively smaller BMD decline with BIC compared to other antiretrovirals [75]. At week 48, mean spine BMD declined by 0.5% with BIC/FTC/TAF versus 1.1% with comparators (p = 0.52), which included both other InSTI (DTG and EVG/c) and PI (ATV/r). Similar findings were observed for hip BMD.

In conclusion, while ART in general has been associated with declines in BMD, treatment with InSTI has been associated with minimal impact on bone health and a favourable safety profile compared to PI based regimens.

## Conclusions

This review focused on the most recent evidence around weight gain and metabolic complications associated with the use of InSTI in PWH, including additional impacts on lipid parameters, glucose metabolism, and bone health. The study results suggest a mild to moderate risk of weight gain associated with the use of InSTI, both in ART naïve and ART experienced PWH, which is highly variable depending on the different sociodemographic and clinical characteristics of the studied population. Certain groups, like women living with HIV, people of Black African ethnicity, those with lower socioeconomic status, and older people, have a greater risk of gaining weight after initiation of InSTI based regimens. The other components of the current ART regimen as well as previous exposure to certain antiretrovirals also have an influence on the degree of weight gain experienced by PWH on InSTI, with less impact on weight with TDF and EFV, and greater weight gain with TAF. However, it is important to note that both the mechanisms behind InSTI-related weight gain and the clinical consequences of weight gain in PWH remain to be clarified.

Regarding the impact of InSTI on metabolic health in PWH, most of the evidence suggests a relatively safe profile of InSTI on lipid parameters and bone health, especially when compared to older ART regimens. On the other hand, recently published studies point towards a greater risk of alterations in glucose homeostasis, including hyperglicaemia, insulin resistance, and diabetes mellitus, associated with the use of InSTI. Both the pathogenetic mechanisms of this association, as well as the clinical implications on morbidity and mortality for PWH remain to be elucidated.

In conclusion, InSTI represent the mainstay of modern ART for the management of PWH, given their high genetic barrier, potency, and good tolerability. The potentially negative impact on metabolic health seems to be mostly influenced by other sociodemographic and clinical factors, although some studies have suggested a direct influence of some 2nd generation InSTI on glucose homeostasis and other metabolic abnormalities. This highlights the importance of adopting an individualized approach to ART in PWH, taking into consideration clinical, sociodemographic factors, and personal preference. There remains a need to further clarify the mechanisms behind metabolic complications associated with the use of InSTI and their clinical implications, in order to focus on prevention and optimal management for a better quality of life in PWH.

## Data Availability

No datasets were generated or analysed during the current study.
